# Karate and Dance Training to Improve Balance and Stabilize Mood in Patients with Parkinson’s Disease: A Feasibility Study

**DOI:** 10.3389/fmed.2017.00237

**Published:** 2017-12-19

**Authors:** Katharina Dahmen-Zimmer, Petra Jansen

**Affiliations:** ^1^University of Regensburg, Regensburg, Germany; ^2^Department of Sport Science, University of Regensburg, Regensburg, Germany

**Keywords:** Parkinson, karate, line dance, standard dance, cognitive function, motor functions, emotional well-being

## Abstract

The present pilot study investigated the effect of karate (according to the rules of the German Karate Federation) and dance training compared to an inactive control group in patients with Parkinson’s disease (PD). 65 patients were recruited. At the end, 37 patients completed the post-test. From those 37 patients, 16 had chosen the karate training, 9 the dance training and 12 the waiting control group. Before and after the whole training phase cognitive performance, emotional well-being and balance were measured. The results showed that both, karate and dance training groups, improved balance. Furthermore, the mood dropped only in the waiting control group receiving no training at all, whereas it remained stable in patients who attended the karate and dance group. The training adherence was higher in the karate than the dance group indicating a high acceptability in PD patients for karate. In sum, karate can have the same positive effects as dance for PD patients. Further studies with larger samples and more rigorous methodologies are required to investigate the reported effects in more detail.

## Introduction

Parkinson’s disease (PD) is determined by the cardinal symptoms bradykinesia, rigidity, tremor, difficulties in balance, and resulting difficulties in gait. This impaired motor performance sometimes has a detrimental effect on the quality of life, because it may be accompanied by emotional disorders like depression or anxiety (1). Up to 57% of PD patients develop cognitive impairments in the first 3–5 years after diagnosis (2). Thus it is evident that PD is a holistic disorder. Furthermore, it is estimated that worldwide, at least four million people in total receive the diagnosis of Parkinson’s disease (3). According to Zhou et al. it is expected that the number of PD patients will rise to the number of 4.94 million in 2030 merely in China (4). Due to this prognosis it is crucial to find alternative therapy concepts to enhance the quality of life of PD patients. One of these concepts includes sports and movement programs.

### Parkinson Disease and Sports

Several studies showed the positive influence of sport training on motor symptoms of PD patients. Shulman et al., for example, proved that endurance as well as strength and flexibility improved by a treadmill, strength and flexibility training (5). Lauzé et al. indicated in a review that physical activity has promising effects on physical capacities (e.g., lower and upper limb strength or endurance) and physical and cognitive functional aspects like for example gait or mobility (6). According to the review of Lauzé et al. (6), psychosocial aspects of life revealed the least potential for an improvement through physical activity. This result is in contrast to a study of Cusso et al. who revealed that non-motor symptoms in PD patients like depression, apathy, fatigue, and sleep disorders were significantly improved by physical activity (7). Most of the 20 studies included in their review used aerobic programs instead of, for example, strength or flexibility programs. Furthermore, due to the different measurement instruments results are difficult to compare across studies. In the present study, two specific movement forms were investigated in more detail, namely, dance and martial arts. Both of them and their possible enhancing effects on different symptoms in PD patients are presented in the following in more detail.

### Parkinson’s Disease and Dance

Dance seems to be an appropriate movement form to be applied in PD patients because it strengthens the muscle of the lower extremities (8) and improves balance which reduces the risk to fall (9). In a meta-analysis regarding the influence of tango argentine on Parkinson’s disease motor systems improved. Furthermore, there was a tendency for life quality to be improved and participation in social activities was augmented (10). In one pilot study, the influence of an unspecific dance training (*N* = 15) was compared to a normal sport training (*N* = 17) and a waiting control group (*N* = 14) (11). The training lasted 60 min for 12 weeks. The dance group improved its motor performance, visual–spatial cognition (reaction time in mental rotation), and emotional parameters (e.g., self-rating depression scale). Summarizing the results, the effect of dance on the patients’ motor systems was stronger than the effect on cognition and emotion.

### PD and Martial Arts

Most studies which showed an improving effect of martial arts on PD are related to TaiChi, an old Chinese martial art. TaiChi includes a series of slow, meditative movements for self-defense, and for the accomplishment of inner peace and calm. Several studies showed a positive effect on motor systems such as balance (12). In another meta-analysis, the authors could not prove an improvement of quality of life (13), a result which was confirmed by a review of Zou and co-workers (4).

Until now, there is no study which investigates the effect of a karate training on PD patients. Karate is a martial art, which entails moving forward and backward while performing arm movements. Up to this point, there are only three studies investigating the effect of karate on cognitive, motor, and emotional symptoms in older people. Muiños and Ballesteros showed an increased dynamic visual acuity in older people who trained karate compared to sedentary controls (14). Visual acuity is a marker of perceptual processing speed. Jansen and Dahmen-Zimmer (15) presented the result that older adults (67–93 years) who just began with the karate training improved their emotional well-being. This result was obtained in comparison to an inactive control group. No cognitive enhancement could be confirmed. In a new study, Jansen et al. found an improvement in subjective mental health and anxiety as well as cognitive processing speed for a karate group, but not a mindfulness-based stress reduction group and a control group (16). In this study 55 healthy participants between the age of 52 and 81 took part and the training lasted for 8 weeks.

### Goal of this Study

It is the first goal of this study to investigate if it is feasible to perform a karate training with PD patients. This provided, we further want to explore if karate as well as dance can improve emotional, cognitive as well as motor performance in patients with PD compared to an inactive control group. Third, by conducting an applied study and by giving the patients the opportunity to choose the kind of movement they like we want to mirror a real-life situation. We expect males to prefer a karate training and females to prefer a dance training.

## Materials and Methods

### Participants

In total, 65 patients diagnosed with idiopathic PD (Hoehn and Yahr stages 1–3) were recruited for participation through a newspaper announcement and with the help of a neurologist. According to our former studies (15), it was our target that 36–45 patients participated until the posttest, 12–15 in each group. The recruitment took place in spring 2016 and the experimental interventions were performed between August 2016 and March 2017. At the end, 37 patients completed the posttest. From those 37 patients, 16 had chosen the karate training, 9 the dance training and 12 the waiting control group. The patients of the waiting control group could not or did not want to attend an experimental group at this time. They were offered to join the karate or dance group after they had completed the intervention. All participating patients denied to actually suffer from the following diseases: osteoporosis, lumbar diseases, cardio pulmonary diseases, or cancer. All patients got consent from their physicians to take part in this study. The three groups did not differ in age, *F*(2,34) = 0.523, n.s., $\eta_{p}^{2}=0.030$, but according to the percentage of females and males, χ^2^(2,*N* = 37) = 8.3, *p* < 0.05. The study was approved by the Ethical committee of the German Association of Psychology. All Patients gave written informed consent according to the ethical declaration of Helsinki. In the intervention groups, some of the attending healthy partners took part (karate group, *N* = 8; dance group, *N* = 4), but only the data of the patients were analyzed.

### Materials

#### Demographic Data

Demographic data were assessed concerning the age, sex, and the physical activity (yes/no). The highest school/university degree was also measured (1 = 10 years of education; 2 = 10 years of education and a further higher education; 3 = university degree). Additionally, the participants were asked to mention any other illnesses and medication in an open text field (see Table 1).

**Table 1:** Demographic data dependent on the training group.

	Karate (*n* = 16)	Dance (*n* = 9)	Control (*n* = 12)	χ^2^ (*df* = 2)	*p*
Sex (male, female)	13, 3	6, 3	8, 4	8.39	0.015
Age (M, SD)	68.87 (7.24)	72.33 (6.69)	70.42 (10.07)	0.523^a^	0.597
Physical activity (yes/no)	7/9	4/5	6/6	0.119	0.942
School education (low/middle/high)	5/4/7	2/6/1	8/4/0	12.52	0.014
Session of training (M, SD)	25.15 (5.48)	20.67 (5.31)		3.89^a^	0.061

*^a^F-statistic of univariate analysis of variance*.

#### Emotional Variables

Subjective well-being was measured with the Multidimensional Mood Questionnaire (17). This measurement includes 24 items with a 5-point rating scale each. The following subscales were integrated: Mood (elevated vs. depressed mood), Fatigue (wakefulness vs. sleepiness), and Alertness (calmness vs. restlessness). Cronbach’s Alpha varied between 0.86 and 0.94.

Anxiety and depression were measured with the Hospital Anxiety and Depression Scale [HADS; (18, 19)] as well as the CEDS Depression Scale (20). The HADS consists of 14 items. Scores under 7 are unremarkable, scores between 8 and 10 are marginal noticeable. Cronbach’s alpha varied between 0.73 for anxiety and 0.78 for depression. With the help of the CEDS the symptoms of fatigue, hopelessness, demotion of oneself, dejection, loneliness, sadness, listlessness, fear, etc. could be analyzed. A score over 23 counts as a sign of a possible depression. Split-half reliability is 0.81.

Subjective health was analyzed with the 12-item Short-Form Health Survey (21), measuring physical as well as mental health. This scale was valid in association with other physical and mental health measurements.

General self-efficacy was investigated with the Short Scale of General Self-Efficacy (22). This scale includes three items with a 5-point rating scale. Cronbach’s alpha varied between 0.81 and 0.86.

#### Cognitive Variables

Cognitive processing speed as well as executive function were measured with the Number Connection Test. In this test, participants have to connect numbers (1–90) printed randomly on a sheet of paper. The test consists of six sheets of paper, two practice sheets and four test sheets. The numbers have to be connected as fast and as accurately as possible in the right order. The mean time of the four test trials is calculated. The test–retest reliability is 0.95 (23).

General cognitive ability was measured with the Parkinson Neuropsychometric Dementia Assessment (PANDA). This scale includes a task of pair association, word fluency, visual–spatial cognition, working memory, delayed retrieval, and assesses mood (24). The five cognitive tests have a specificity of 91% and a sensitivity of 77% for patients with Parkinson’s disease. In this study, we focused on the analysis of the cognitive score.

#### Motor Variables

Balance was measured with the one-leg stand. Here, patients were required to stand on one leg as long as possible, but at least for 60 s. Patients should cross their arms in front of their chests and fix some point in the room. The experimenter took the time the patients were able to stand on one leg. The best of three trials was taken for the analysis (25).

### Procedure

Each training took place once a week for 1 h and in a separate room. The whole trainings phase lasted for 30 weeks. The trainers registered the training attendance of each patient and the attending partner. The mean attendance of the dance training was 20.67 h and the one of karate training 25.15, see Table 1.

### Deutscher Karate Verband (DKV) Karate

The karate group received a Shotokan karate training according to the German Karate Federation. This kind of training involves the elements of Kihon, Kumite, and Kata. The training began with a short warm-up followed by the learning and exercising of some specific arm and leg movements (Kihon), sessions with a partner (Kumite), and the learning of sequences of movements (Kata). Kata are the most elaborated exercises because different movements have to be remembered in a prescribed order. The training further involved breathing exercises, strong and soft, and slow and fast movements. It ended with a short relaxation phase. There were three experienced trainers (two males, one female) with more than 20 years of practice and more than 10 years of teaching experience. They rotated in their teaching activities.

### Dance

The dance sessions included simple dance movements which were compounded to a choreography and danced together in a line, i.e., elements of line dance were integrated. Later, standard dance forms such as rumba and waltz, were applied. Three different dance trainers (two males and one female) with several years of experience taught the dance lessons. They rotated in their teaching activities.

The rotation of trainers was welcomed by all patients in both groups. The tests were applied 1 week before the specific training started and again 1 week after the last training session. Each test session lasted around on hour. The tests were conducted by psychological research assistants.

### Statistical Analysis

The categorical data were analyzed with chi-square tests, see Table 1. To investigate the relation between cognitive, emotional, and motor performance, a correlation analysis was conducted independent of group with only the most relevant cognitive, motor, and emotional tests: the cognitive score of the PANDA, the results of the depression scale and the self-efficacy, and the performance in the one-leg stand. For metric-dependent variables, analyses of variance (ANOVAs) were performed. Group served as a between-subjects factor (KG, DG, WKG) and time (pretest and posttest) as a within-subject factor. Thus, for the investigation of the effect of karate or dance training compared to the waiting control group on emotional well-being, cognitive functioning, and balance, we conducted 3 × 2 repeated-measures ANOVAs. Significance levels were set at ɑ = 0.05. Because one focus of the study lay on the feasibility of karate and dance in PD patients and due to clarity reasons, we will present only the (marginally) significant results in the following Section “Results.”

## Results

### Adherence Rate

In the karate group, there were 25 patients at the beginning and 16 completed the posttest. Nine patients did not complete the study due to private reason (1), health complaints (4), time constraints (2), death (1), or relocation (1). In the dance training group, 12 patients started with the training and 3 of them left it before the posttest due to health complaints (1), time constraints (1), and no interest (1). From the waiting control group, 18 patients completed the pretest but only 12 appeared again to complete the posttest. The remaining six patients did not give any reason for not showing up.

### Correlational Analysis

The correlational analysis showed a significant positive relation between the performance in the one-leg stand and the self-efficacy (*r* = 0.398, *p* = 0.015) and a negative relation between the balance performance and the depression value (*r* = −0.407, *p* = 0.010). A better balance performance is related to a higher self-efficacy and a minor depression rate.

### Emotional Well-being

#### Subjective Well-being

##### MDBF Mood

**Figure 1:**
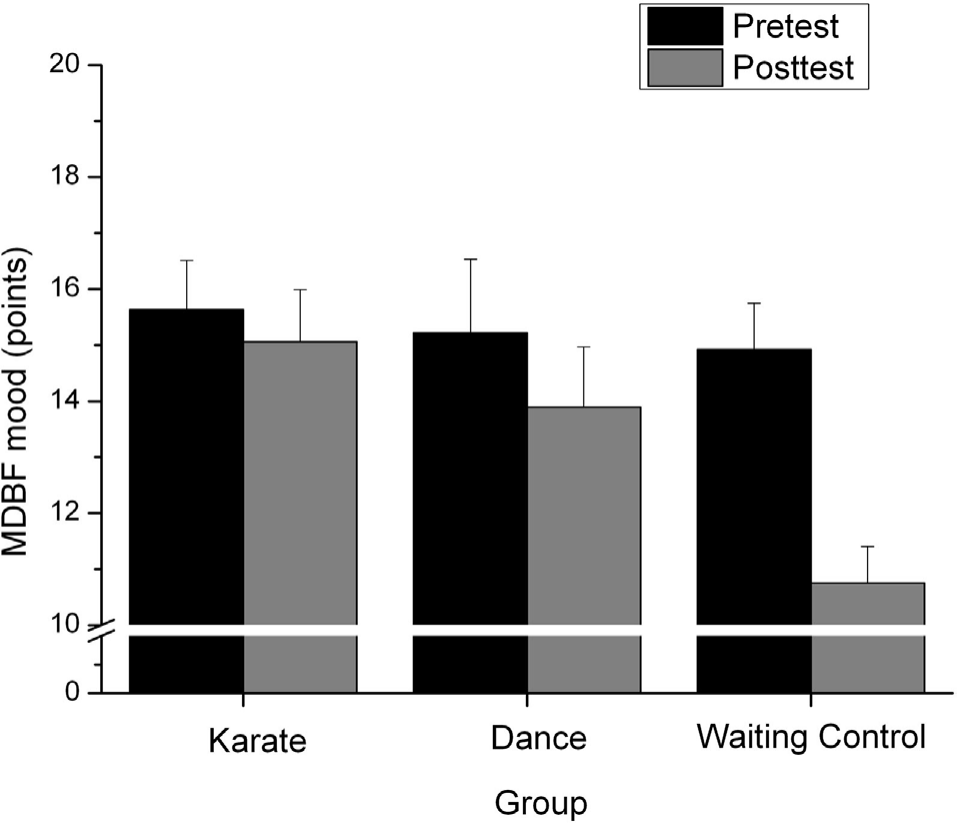
Points in the mood measurement in the MDBF (in the pretest and posttest) for the karate, dance and waiting control group.

The repeated-measure analysis yielded a significant main effect of “time,” *F*(1,34) = 12.29, *p* = 0.001, $\eta_{p}^{2}=0.266$, a marginally significant effect of the factor “group,” *F*(2,34) = 2.67, *p* = 0.083, $\eta_{p}^{2}=0.136$ and a significant interaction between both factors, *F*(2,34) = 4.003, *p* = 0.027, $\eta_{p}^{2}=0.191$. The one-tailed *t*-test for independent samples of the difference score between posttest and pretest between KG and WCG showed a significant minor degradation of the mood from post compared to pretest, *t*(22.17) = 2.58, *p* = 0.08 and a minor degradation between DG and WCG, *t*(18.98) = 1.97, *p* = 0.031, see Figure 1. Significance level was set to *p* = 0.025 because of multiple testing.

The MDBF subscales fatigue and agitation did not receive any significant result.

### Depression and Anxiety

Concerning the analysis of the *HADS* and the subscale of depression, the repeated-measure analysis yielded only a significant effect of the factor “group,” *F*(2,34) = 3.86, *p* = 0.031, $\eta_{p}^{2}=0.185$. Bonferroni-corrected multiple comparisons indicated that the KG group (M = 4.87, SD = 2.96) had a smaller score than the WCG (M = 7.62, SD = 2.2) (*p* = 0.031). There was no effect in relation to the DG (M = 6.67, SD = 2.61). The result of the HADS depression subscale was confirmed by the *ADS analysis*, yielding only a significant effect of the factor “group,” *F*(2,34) = 3.97, *p* = 0.028., $\eta_{p}^{2}=0.189$. Bonferroni-corrected multiple comparisons showed that the KG group (M = 13.12, SD = 7.06) received a smaller score than the WCG (M = 18.95, SD = 4.24, *p* = 0.064) and the DG (M = 19.16, SD = 7.24, *p* = 0.084). There was no difference between the DG and WCG. The HADS subscale of anxiety revealed no effect at all.

### Subjective Health (SF-12)

For the physical score, there was no significant main effect at all. The analysis of the mental score yielded only a significant effect of the factor “group,” *F*(2,34) = 4.85, *p* = 0.014, $\eta_{p}^{2}=0.051$. Bonferroni-corrected multiple comparisons showed that the KG group (M = 46.25, SD = 9.97) showed a higher score than the WCG (M = 35.39, SD = 7.54) (*p* = 0.012). There was no effect in relation to the DG (M = 40.07, SD = 9.98).

### Self-Efficacy

The results showed simply a significant group effect, *F*(2,34) = 9.47, *p* = 0.001, $\eta_{p}^{2}=0.358$. Bonferroni-corrected multiple comparisons revealed that the KG group (M = 4.14, SD = 0.51) showed a higher score than the WCG (M = 3.22, SD = 0.82), (*p* = 0.003) and the DG (M = 3.09, SD = 0.72, *p* = 0.002) and no difference between WCG and DG (*p* = 1.0).

### Cognitive Performance

Regarding cognitive processing speed and executive functions, there was just one significant main effect of the factor “time,” *F*(1,34) = 4.795, *p* < 0.01, $\eta_{p}^{2}=0.124$. Patients in each group showed a better performance in the posttest compared to the pretest.

Concerning the analysis of the PANDA, there was just one significant main effect of the factor “time,” *F*(1,34) = 8.312, *p* < 0.01, $\eta_{p}^{2}=0.196$. Patients in each group showed a better performance in the posttest compared to the pretest.

### Motor Performance

**Figure 2:**
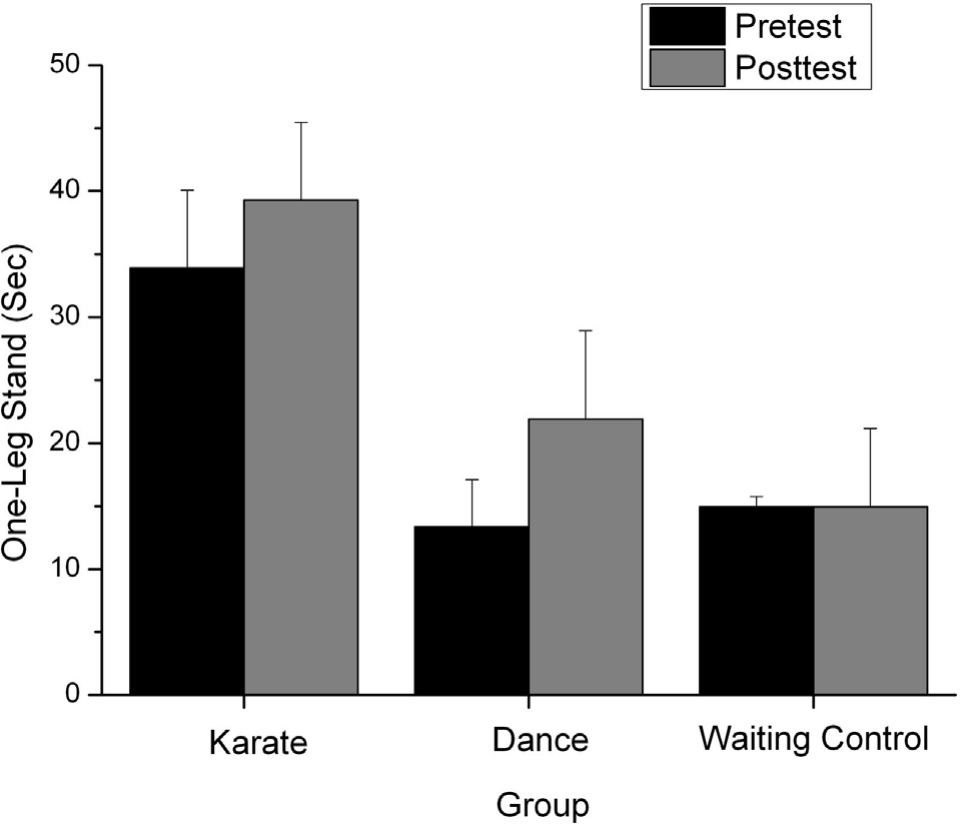
Performance in the one-leg stand (measured in seconds) in the (in the pretest and posttest) for the karate, dance, and waiting control group.

The analysis in the performance of the one-leg stand revealed a significant main effect of the factor “time,” *F*(1,34) = 6.615, *p* < 0.05, $\eta_{p}^{2}=0.163$. Patients in each group showed a better performance in the posttest compared to the pretest. There was also a significant main effect of the factor “group,” *F*(2,34) = 4.031, *p* < 0.05, $\eta_{p}^{2}=0.192$ and a marginally significant interaction between both factors, *F*(2,34) = 2.881, *p* = 0.07, $\eta_{p}^{2}=0.045$, see Figure 2. The analysis of one-tailed *t*-test for independent samples of the difference score (posttest–pretest) between KG and WCG, *t*(19) = 2.37, *p* = 0.02 and DG and WCG, *t*(26) = 2.18, *p* = 0.02 showed a significant improvement of both experimental groups compared to the waiting control group. Significance level was set to *p* = 0.025 due to multiple testing.

The one-leg stand test was performed for mostly 60s, and thereafter terminated. In the KG, already 37.5% participants achieved this score in the pretest. No participant of the DG and 16.7% of the WCG achieved this score. Because of this “ceiling effect,” no additional improvement could be measured for these persons in the posttest.

## Discussion

Regarding our first goal, it was shown that it is possible to conduct karate with PD patients. Furthermore, karate seemed to be more attractive for male patients compared to females: more males chose to attend a karate session compared to a dance session. Furthermore, the self-selection of the type of sport resulted in the fact that more patients with a higher school degree chose karate, an effect which might be mediated by the higher attendance of males in this group. Moreover, the karate group showed higher scores in some of the pretests than the other groups. Especially in the one-leg stand test more than one third of the karate participants started with the highest score possible. Therefore, it is impossible to demonstrate their improvement (ceiling effect). Concerning the results of this feasibility study, no difference between the effect of karate or dance on emotional, cognitive, or motor performance was found. Both interventions showed an improvement in balance as well as a prevention of mood drops. Another interesting result was that emotional, cognitive, and motor parameters relate to each other, suggesting a holistic approach when dealing with treatment of PD.

In addition, the study clearly proves a high treatment adherence, especially in the karate training, which might be an indicator for the high acceptability in PD patients. Because other kinds of therapies, such as long-term medication, suffer from a lower adherence (26), the karate training has the potential to be accepted as a complementary therapy. This is the first study to provide evidence that karate is an appropriate training to improve balance in PD patients. This is in line with the claim of Morgan and Fox that exercise is increasingly recognized as important tool to combat impairment of motor systems in PD patients (27). Furthermore, compared to the inactive control group, no decline of emotional well-being took place in the karate group. This result relates to the karate study with older people showing a training effect of karate on self-related mental health (16) and indicates that in PD patients karate may also be appropriate to maintain emotional well-being while living with PD. One reason for this might be that karate gives the opportunity to train in a group with respect, and without competition, which maintains the highly relevant feeling of social support. As another likely reason self-esteem could improve. Moreover, an enhancement of some spiritual insight could result in calmness of mind. The enhancement of the spiritual insight is in line with positive effects of mindfulness training in clinical interventions (28).

### The Influence of Dance on Balance and Mood in PD Patients

The specific dance training applied in this study, a combination of line dance and standard dance, showed an improvement in balance as well as a stable state of emotional well-being compared to the inactive waiting control group. This is in accordance with Earhart’s claiming that dance can be a clinically meaningful therapy for individuals with PD (29). This result is also in line with the meta-analysis investigating the effects of argentine tango on PD patients, demonstrating the positive effect on motor behavior (10). One benefit of dance might be the effect of cueing, which is mostly auditory, but can also be visual or somatosensory. With the help of this cueing the movement gets facilitated. This is in accordance with studies showing that rhythmic auditory stimulation is helpful to improve walking performance in patients with PD (30). Furthermore, the stable state of emotional well-being found in this study confirms the results of Lewis et al. indicating an overall reduction on emotional discomfort (31).

### No Effects on Cognition for Karate and Dance

In contrast to some other studies, for example the dance study of Natale et al., we did not find any significant improvements for cognitive functions (32). One reason for this might be specific tests used in our study, i.e., the PANDA and some sort of trail making test for cognitive speed and executive functions. In other studies showing positive effects, executive functions are investigated in more detail (32). The non-significant effects of karate training on cognition are in line with the study of Jansen and Dahmen-Zimmer in older people (mean age: 78 years), but contradict the study of Jansen et al. (16), who found an improvement in the processing speed of older people (mean age: 63 years). Both studies differ in the mean age of the participants, so we might tentatively assume that an improvement of cognition with a Karate training is easier to achieve with participants before the end of their professional life. Concerning the positive effect of dance on cognition, Kattenstroth et al. report an improvement on cognitive functions after healthy individuals (mean age: 68 years) joined a dance training for six months (33). Again, the individuals were healthy and under 70 years old.

### Limitation and Chances

The present investigation serves as a pilot and feasibility study exploring the potential benefits of karate as another promising exercise tool in the treatment of PD. The study is limited by the fact, that a randomization is missing, because the patients were free to choose the form of exercise they liked to do. This experimental setting was selected to enhance the validity of the study. It is quite unlikely that patients might be motivated to take part committedly in a motor training they do not like. One might suspect that this would result in a high number of drop-outs and/or and unreliable results. On the other hand, we could not exclude that the obtained effects are biased by a preference effect. We gained interesting information, that is males like to choose karate while females chose dance. Also, the patients who choose karate seem to have a better physical and mental health as the patients of the other two groups. The statistical analysis was impeded by these facts. It is also uncommon to invite the partners to take part in the intervention group. This limited internal validity but raised ecological validity. In this manner, a reasonable compromise between a high internal and external validity was implemented in this study. Another limitation is the fairly small number of participating patients.

### Future Research and Clinical Practice

This is the first study showing that karate might be a positive treatment method for the emotional, cognitive, and motor symptoms of PD patients. The advantage of this study lies in the investigation of the impact of karate and dance on body, emotion, and cognition. However, more studies with a bigger sample size and clear randomization have to be conducted. It may also be useful to investigate the effect of different karate postures as well as the role of the patients’ partners in the dance group in more detail.

Concerning the clinical practice, we can assume that it is useful for the PD patients to choose the physical training they like the most. If they get the general allowance of their neurologist, they are able either to attend an adapted karate or a dance training. Both adapted training forms should be considered as non-medicinal additional therapy beside medicinal treatment.

## Ethics Statement

The study was approved by the Ethical committee of the German Association of Psychology, spring 2016.

## Author Contributions

KD-Z: conception of the work; acquisition and interpretation of the data: surveillance of the interventions; drafting the work, revising it critically; final approval of the version to be published; agreement to be accountable for all aspects of the work. PJ: conception of the work; analyzing and interpretation of the data; drafting the work, revising it critically; final approval of the version to be published; agreement to be accountable for all aspects of the work.

## Conflict of Interest Statement

The authors declare that the research was conducted in the absence of any commercial or financial relationships that could be construed as a potential conflict of interest.
